# Supplementary material for: Ontogeny independent expression of LPCAT2 in granuloma macrophages during experimental visceral leishmaniasis
Source: Commun Biol. 2026 Mar 20;9:641. doi: 10.1038/s42003-026-09904-4 (PMC13168283; doi:10.1038/s42003-026-09904-4)
Supplement: Supplementary file 2 — Supplementary Information [file 42003_2026_9904_MOESM2_ESM.pdf]

Ontogeny independent expression of LPCAT2 in granuloma macrophages during experimental visceral leishmaniasis

Shoumit Dey[1†], Jian-Hua Cao[2†], Benjamin Balluff[2], Gaia Mazza[1], Helen Ashwin[1], Lesley Gilbert[3], Sally James[3], Adam A. Dowle[3], Grant Calder[3], Nidhi Sharma Dey[1], Peter O'Toole[3], Ron M. A. Heeren[2*,] Paul M. Kaye[1*]

[1] York Biomedical Research Institute, Hull York Medical School, University of York, York, United Kingdom

[2]Maastricht MultiModal Molecular Imaging (M4I) Institute, Division of Imaging Mass Spectrometry, Maastricht University, Maastricht, the Netherlands

[3] Biosciences Technology Facility, Department of Biology, University of York, York, United Kingdom

[†] These authors contributed equally

*** Correspondence:**

Paul Kaye and Ron Heeren

paul.kaye@york.ac.uk (ORCID: 0000-0002-8796-4755); r.heeren@maastrichtuniversity.nl (ORCID: 0000-0002-6533-7179)

Keywords: leishmaniasis, immunometabolism, infectious disease, Lands Cycle, granulomas, spatial multi-omics

Number of supplementary figures: 9

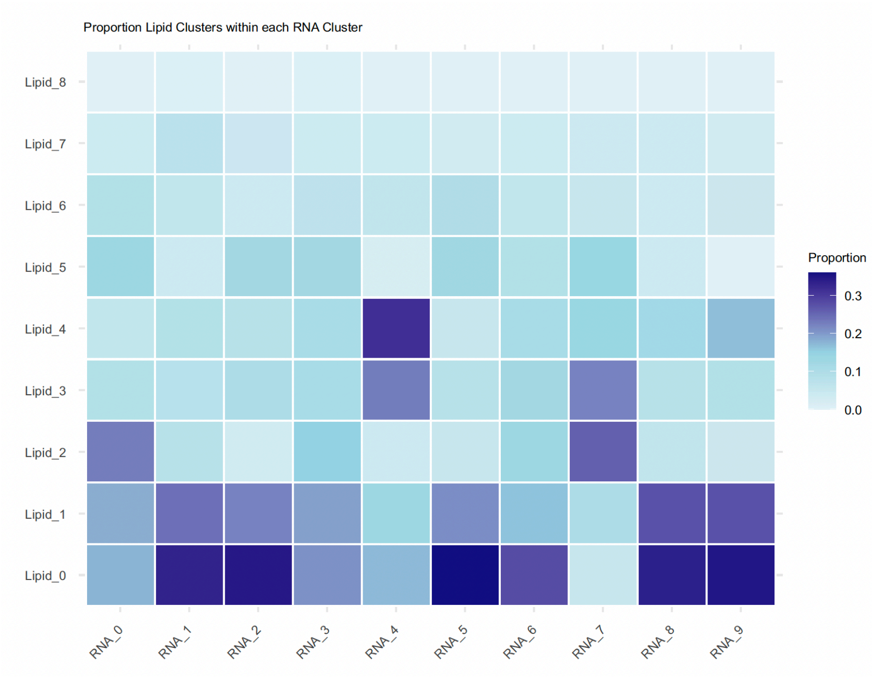

Fig. S1: Heatmap showing a proportion of RNA and Lipid cluster overlaps in the integrated spatial Visium and Lipidomic spots across naïve and infected mice.

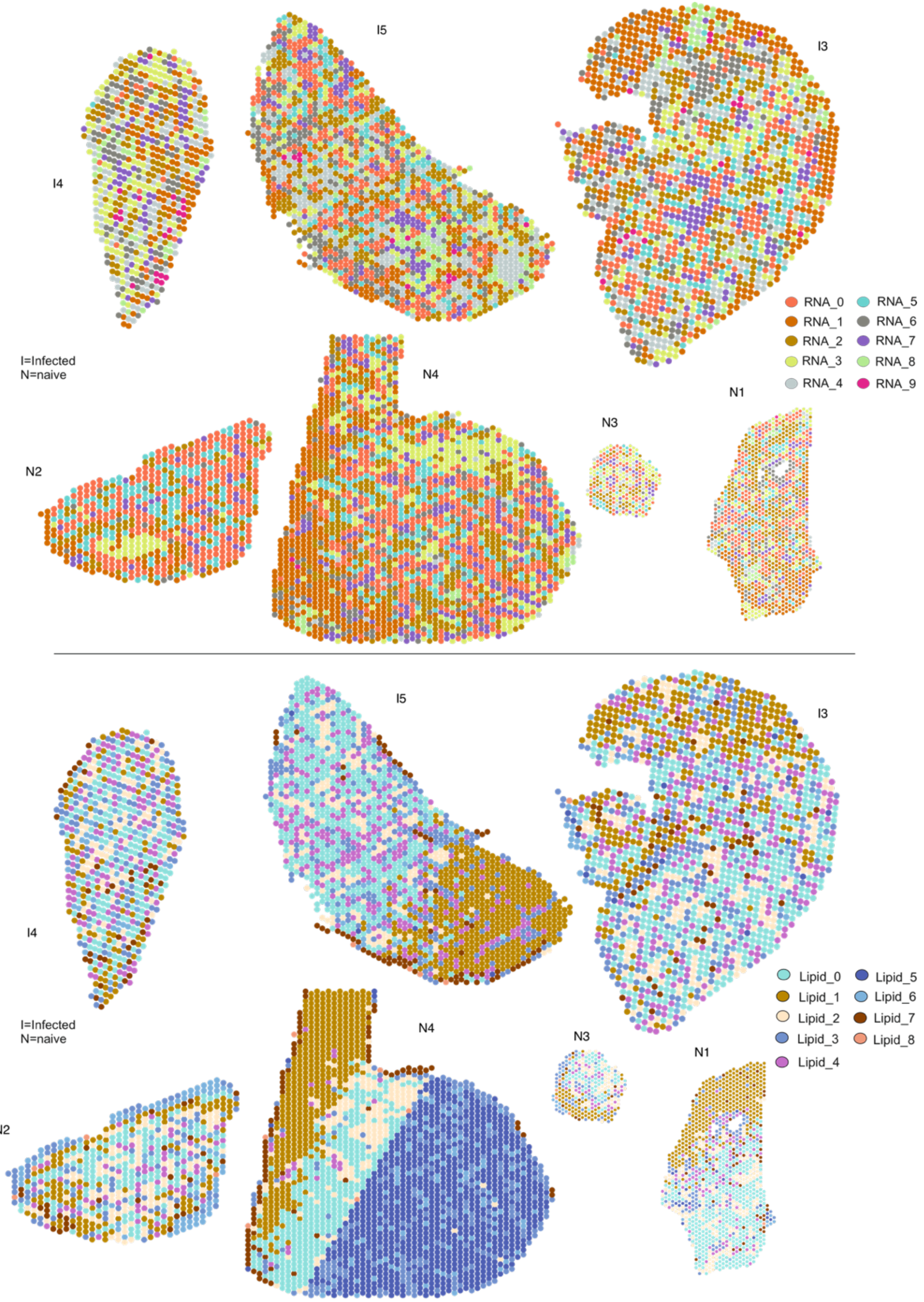

Fig. S2: Spatial plot showing a representative infected section with spatial spots coloured by their cluster colour (RNA clusters - top and Lipid clusters - bottom)

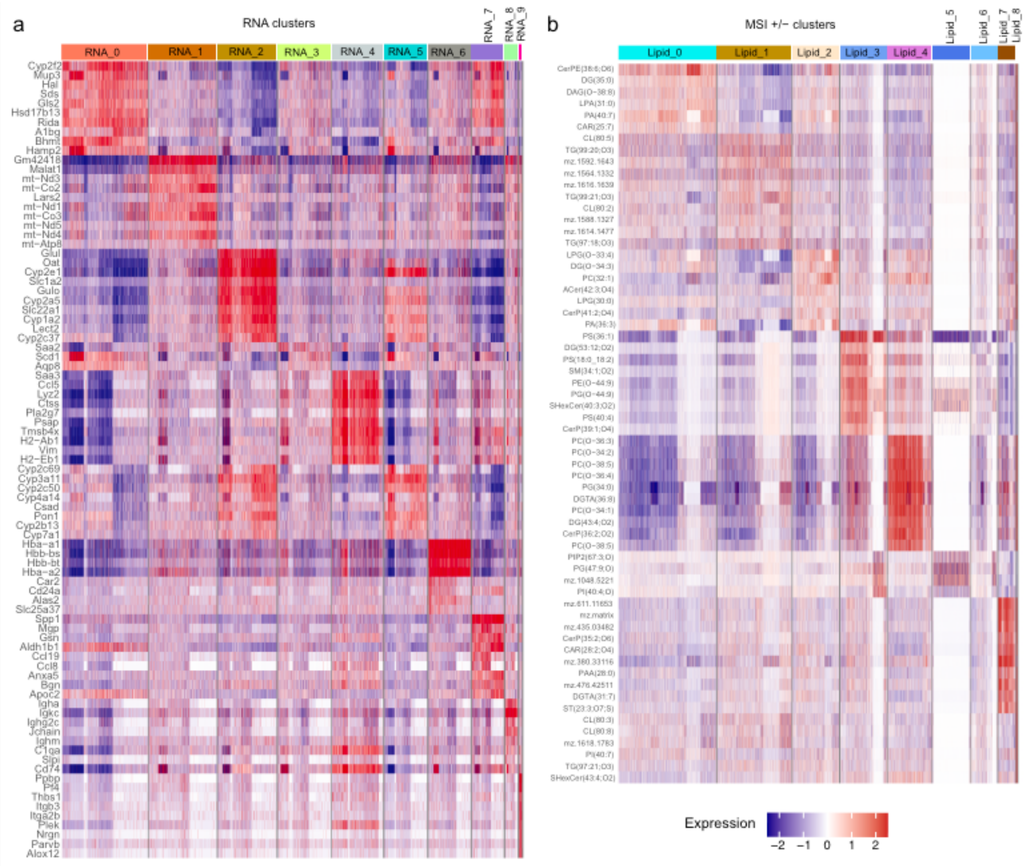

Fig. S3: Heatmap showing differential transcription **A**) and lipid species **B**) between RNA_clusters and Lipid_clusters respectively

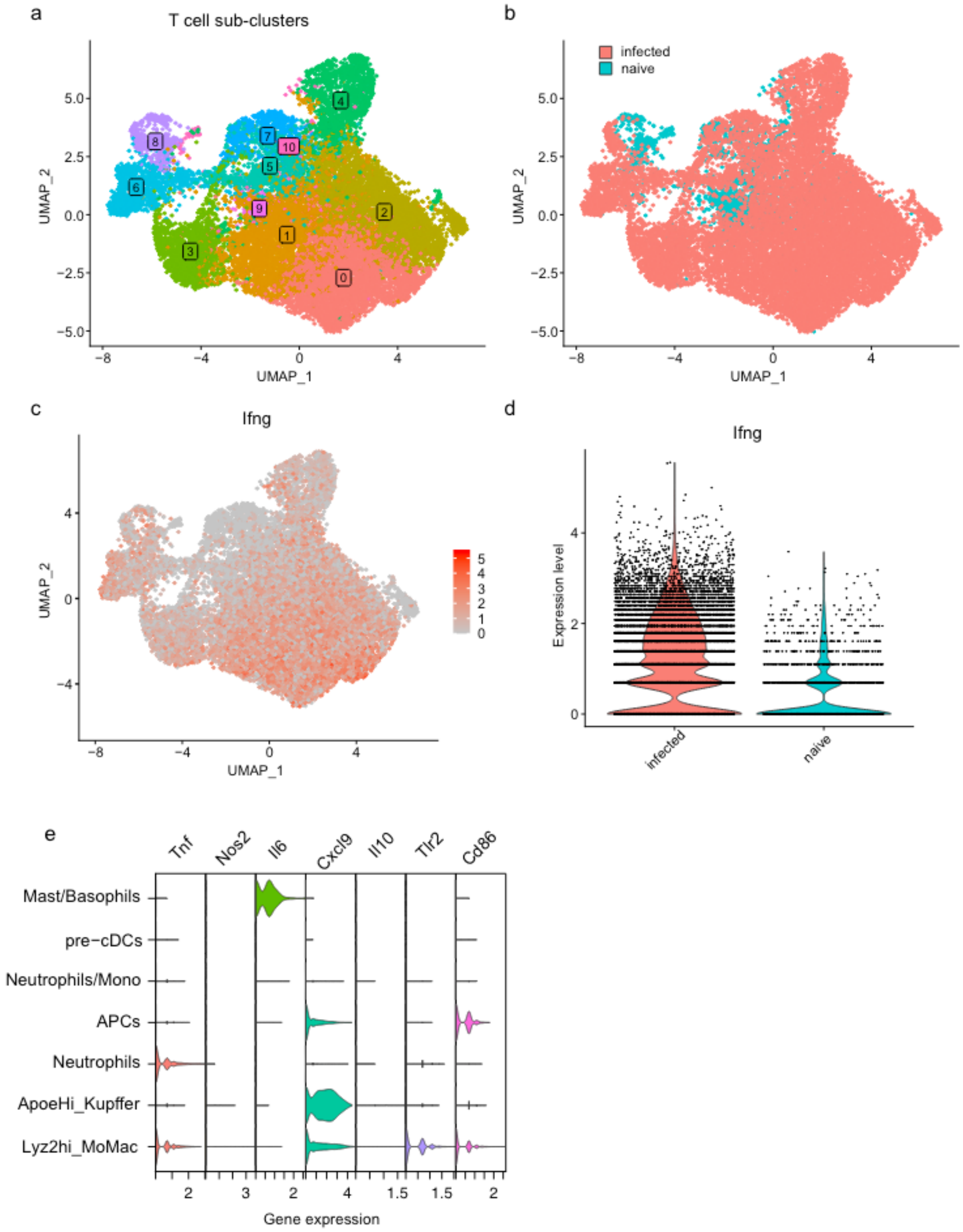

Fig. S4: **a-c**) Single-cells shown on 2-dimensional UMAP scatter plots coloured by T-cell sub-clusters (a), sample origin (b) and *Ifng* expression shown as normalized transcripts (c). **d**) Violin plots showing *Ifng* expression between naïve and infected mice. **e**) Stacked violin plot showing expression of *Tnf*, *Nos2*, *Il6*, *Cxcl9*, *Il10*, *Tlr2*, *Cd86* across myeloid populations

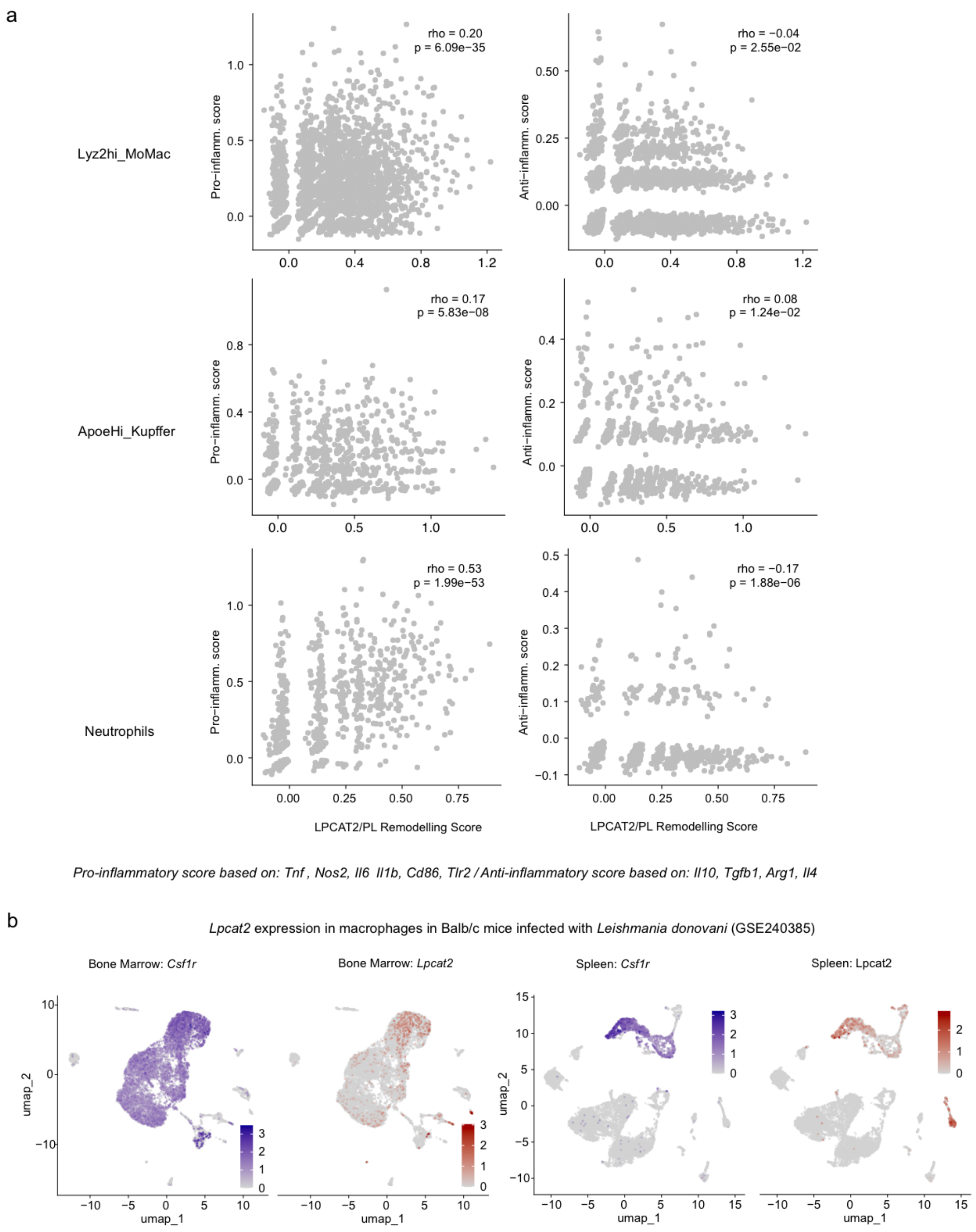

Fig. S5: **a**) Scatter plots showing Spearman's correlation (between LPCAT2/PL Remodelling Score and either Pro-inflammatory score (left panels) or Anti-inflammatory score (right panels), for Lyz2HiMoMac (top), ApoeHi_Kupffer (middle) and Neutrophils (bottom). **b**) Scatter plots on UMAP axes depicting macrophage marker *Csf1r* and *Lpcat2* expression bone marrow and spleen of *Leishmania donovani* infected Balb/c mice from publicly available resource GSE240385.

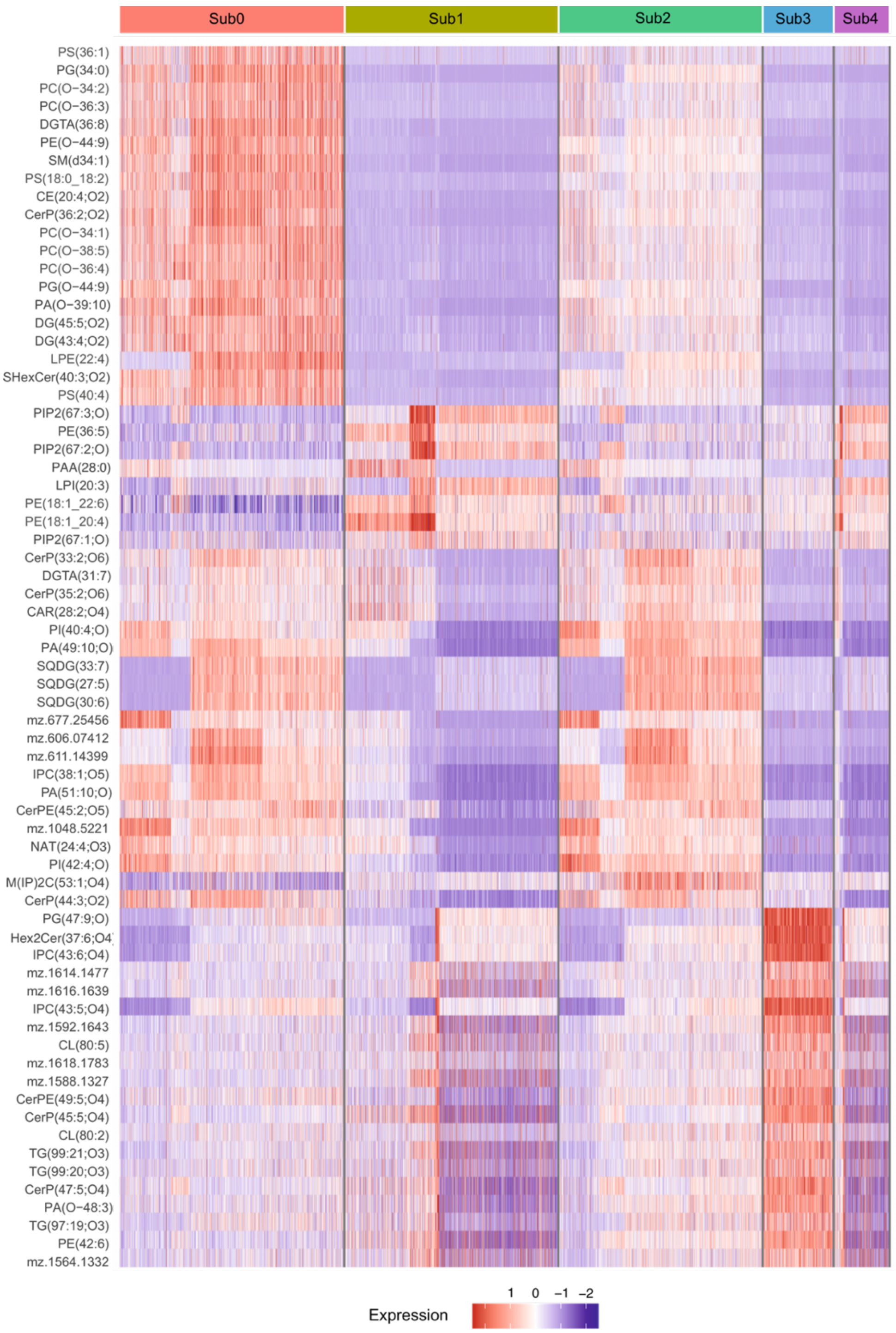

Fig. S6: Heatmap showing differential lipid species between sub-clusters of RNA0/4/7 i.e. Sub0, Sub1, Sub2, Sub3, Sub4. Sub0 and Sub2 are groups specific to infection only and Sub0 represent immune granulomas.

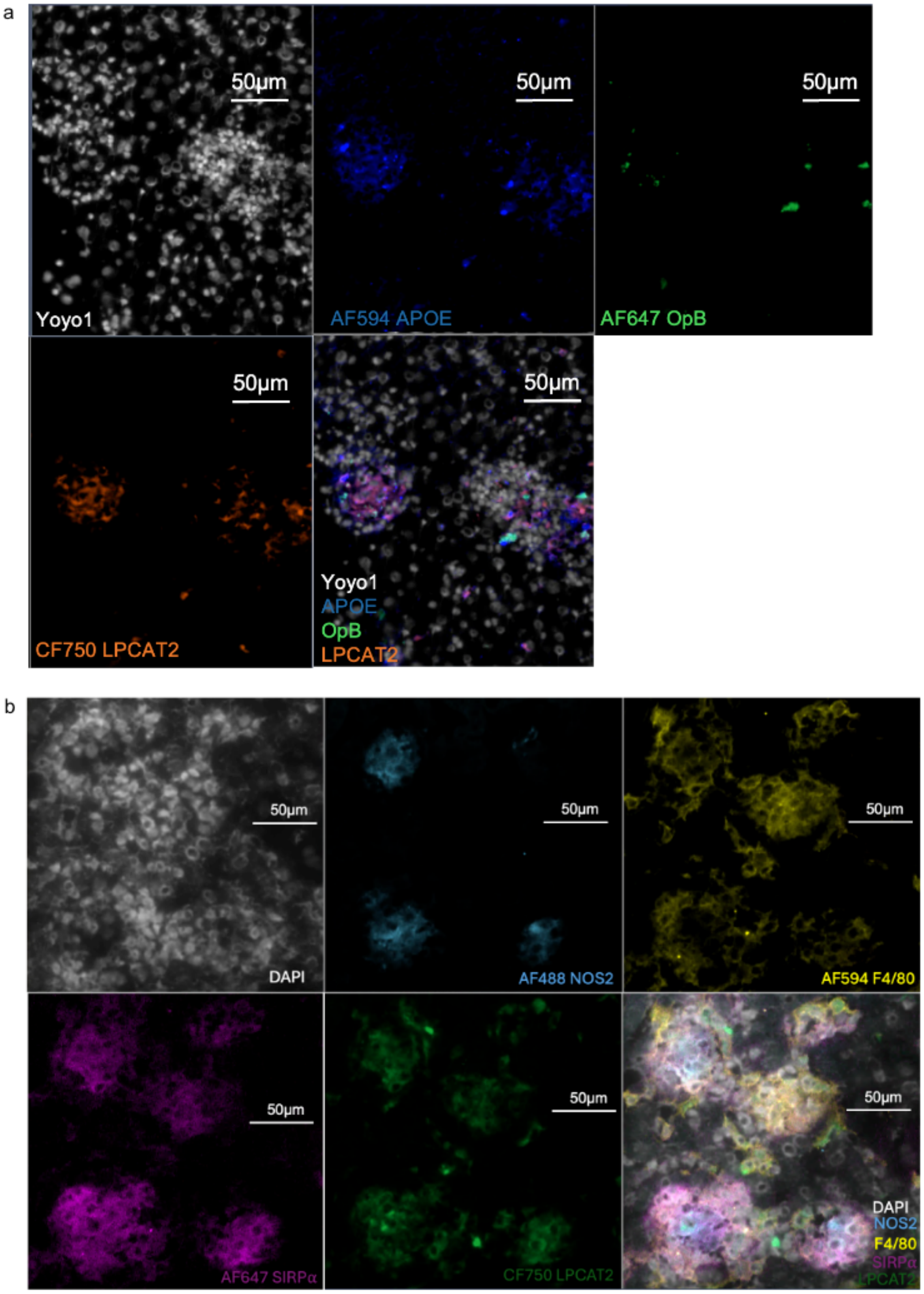

Fig. S7: a) Immunohistochemistry image showing nucleus counter stained with Yoyo-1 along with antibody staining with LPCAT2 (CF750), ApoE (AF594) and OpB(AF647) infected mouse (representative) split into all channels and composite **b**) Same as A but for DAPI (nuclear staining), NOS2 (AF488), F4/80 (AF594), SIRP-α (AF647) and LPCAT2 (CF750).

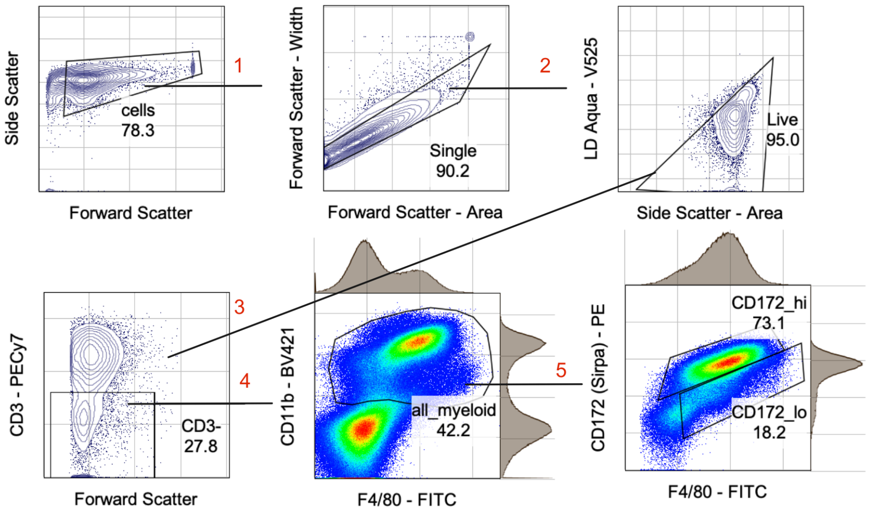

Fig. S8: Sorting strategy for proteomics on CD172hi versus CD172low CD11b+F4/80+ cells from infected liver showing cells selected using forward and side scatter, single cells using forward and side scatter, live cells, CD3- and then CD11b+F4/80+ cells selected as those expressing high CD172 or not

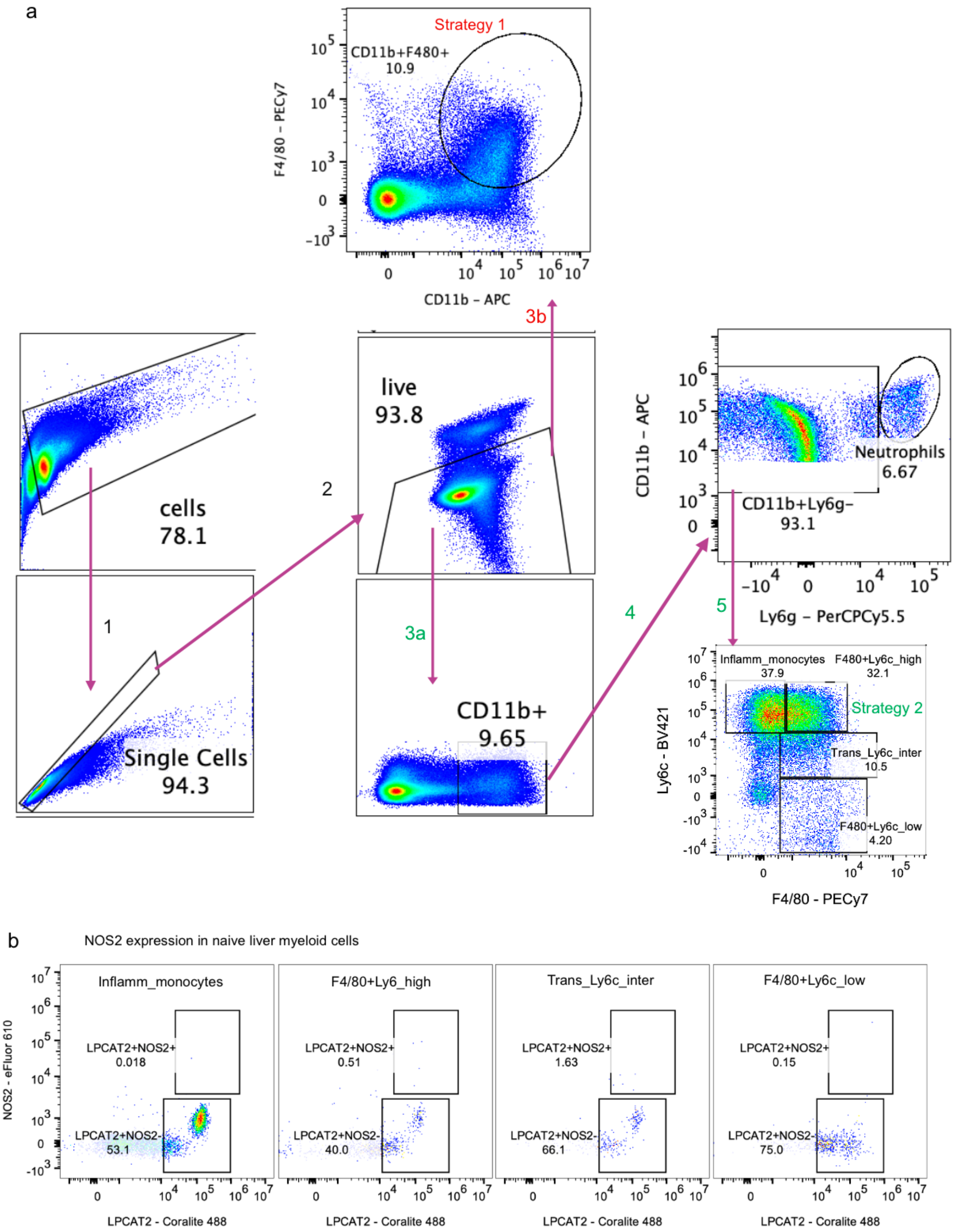

Fig. S9: a) Flow cytometry gating strategy for identifying liver myeloid cell populations. Sequential gating shows: initial cell population based on forward/side scatter, (1) single cell discrimination, (2) live cells, (3a) CD11b+F4/80+ (strategy 1) Or (3b) CD11b+ cells, (4) neutrophil exclusion (CD11b+Ly6g-), and (5) final myeloid subpopulations based on F4/80 and Ly6c expression as strategy 2. Strategy 2 (bottom right) classifies myeloid cells into four populations: inflammatory monocytes, F4/80+Ly6c_high, transitional Ly6c_intermediate, and F4/80+Ly6c_low resident macrophages. **b**) NOS2 expression analysis in naive liver myeloid cell subsets as per strategy 2 in A. Flow cytometry plots show LPCAT2 vs NOS2 expression in four myeloid populations as in A. Numbers indicate percentage of LPCAT2+NOS2+ (top) and LPCAT2+NOS2- (bottom) cells within each population.
