# Supplementary material for: Ontogeny independent expression of LPCAT2 in granuloma macrophages during experimental visceral leishmaniasis
Source: Commun Biol. 2026 Mar 20;9:641. doi: 10.1038/s42003-026-09904-4 (PMC13168283; doi:10.1038/s42003-026-09904-4)
Supplement: Supplementary file 3 — Description of Additional Supplementary Files [file 42003_2026_9904_MOESM3_ESM.pdf]

Description of Additional Supplementary Files

File name: Supplementary Data 1

Description: mz identifications

File name: Supplementary Data 2

Description: scRNA cell type markers

File name: Supplementary Data 3

Description: subcluster lipid markers

File name: Supplementary Data 4

Description: CD172hi vs CD172lo proteins

File name: Supplementary Data 5

Description: CD172hi enrichment
