# Supplementary material for: Ontogeny independent expression of LPCAT2 in granuloma macrophages during experimental visceral leishmaniasis
Source: Commun Biol. 2026 Mar 20;9:641. doi: 10.1038/s42003-026-09904-4 (PMC13168283; doi:10.1038/s42003-026-09904-4)
Supplement: Supplementary file 9 — Reporting summary [file 42003_2026_9904_MOESM9_ESM.pdf]

Corresponding author(s): Ron MA Heeren, Paul M Kaye

Last updated by author(s): Feb 15, 2026

Reporting Summary

Nature Portfolio wishes to improve the reproducibility of the work that we publish. This form provides structure for consistency and transparency in reporting. For further information on Nature Portfolio policies, see our Editorial Policies and the Editorial Policy Checklist.

Statistics

For all statistical analyses, confirm that the following items are present in the figure legend, table legend, main text, or Methods section.

n/a	Confirmed	
☐	☒	The exact sample size (*n*) for each experimental group/condition, given as a discrete number and unit of measurement
☐	☒	A statement on whether measurements were taken from distinct samples or whether the same sample was measured repeatedly
☐	☒	The statistical test(s) used AND whether they are one- or two-sided<br>*Only common tests should be described solely by name; describe more complex techniques in the Methods section.*
☒	☐	A description of all covariates tested
☐	☒	A description of any assumptions or corrections, such as tests of normality and adjustment for multiple comparisons
☐	☒	A full description of the statistical parameters including central tendency (e.g. means) or other basic estimates (e.g. regression coefficient) AND variation (e.g. standard deviation) or associated estimates of uncertainty (e.g. confidence intervals)
☐	☒	For null hypothesis testing, the test statistic (e.g. *F*, *t*, *r*) with confidence intervals, effect sizes, degrees of freedom and *P* value noted<br>*Give P values as exact values whenever suitable.*
☒	☐	For Bayesian analysis, information on the choice of priors and Markov chain Monte Carlo settings
☒	☐	For hierarchical and complex designs, identification of the appropriate level for tests and full reporting of outcomes
☒	☐	Estimates of effect sizes (e.g. Cohen's *d*, Pearson's *r*), indicating how they were calculated

Our web collection on statistics for biologists contains articles on many of the points above.

Software and code

Policy information about availability of computer code

Data collection	For data collection, various specialized software programs were employed. For spatial transcriptomics, 10x Genomics SpaceRanger(1.3.0) was used to align sequencing data to the mouse reference genome (mm10) and to generate spatially-resolved gene count matrices. Mass spectrometry imaging (MSI) data were collected using FlexImaging v5.0 and SCiLS Lab (version 2023b), with additional lipid identification analysis performed via LipoStarMSI (edition 2023). Single-cell RNA sequencing data collection utilized the Cell Ranger software (version 6.1.0) from 10x Genomics to align sequencing reads and generate gene-barcode matrices. Proteomics data were acquired using Compass HyStar software (version 6.2, Bruker) for mass spectrometry. Additionally, imaging data acquisition involved Leica Aperio CS2 scanner software for histological analyses.
Data analysis	For data analysis, a combination of commercial, open-source, and custom software was employed. MALDI mass spectrometry imaging data were analyzed using SCiLS Lab software (version 2023b), while lipid identification was performed with LipoStarMSI software (edition 2023), interfaced with the Lipid Maps database. Single-cell RNA sequencing and spatial transcriptomics data were processed using Cell Ranger software (version 6.1.0) from 10X Genomics, followed by downstream analyses using the Seurat R package (version 4.3.0). Integrated spatial transcriptomics and mass spectrometry imaging analyses were facilitated by custom scripts implemented in R (version 4.1.0). Cell-type abundances and spatial predictions were estimated using the Cell2location. Data-independent acquisition (DIA) proteomics data analysis was performed using DIA-NN software (version 1.8.2.27), with differential protein expression analysis carried out in FragPipe-Analyst utilizing the limma package. All code related to analysis is publicly available on GitHub (https://github.com/jipsi/spatial_lipid_gene).

For manuscripts utilizing custom algorithms or software that are central to the research but not yet described in published literature, software must be made available to editors and reviewers. We strongly encourage code deposition in a community repository (e.g. GitHub). See the Nature Portfolio guidelines for submitting code & software for further information.

Data

Policy information about availability of data

All manuscripts must include a data availability statement. This statement should provide the following information, where applicable:

- Accession codes, unique identifiers, or web links for publicly available datasets
- A description of any restrictions on data availability
- For clinical datasets or third party data, please ensure that the statement adheres to our policy

Data Availability
Spatial and single-cell transcriptomic data available on gene expression omnibus with the accession codes GSE290324 and GSE290325 respectively. Proteomic mass spectrometry data sets and results files are referenced in ProteomeXchange (PXD058137) and available to download from MassIVE (MSV000096486) [doi:10.25345/C5ST7F84G]. Raw data for Figure 6 (per granuloma cell detection) and Figure 8 (FCS files, cell proportions and counts) are available at 10.6084/m9.figshare.30880784 and 10.6084/m9.figshare.30880754 respectively.
Code Availability
All related code and instructions are available on https://github.com/jipsi/spatial_lipid_gene with processed Rds files available on Zenodo (Dey, S. RDS files for Ontogeny independent expression of LPCAT2 in granuloma macrophages during experimental visceral leishmaniasis. doi.org/10.5281/zenodo.18636666 (2026))

Research involving human participants, their data, or biological material

Policy information about studies with human participants or human data. See also policy information about sex, gender (identity/presentation), and sexual orientation and race, ethnicity and racism.

Reporting on sex and gender	*Use the terms sex (biological attribute) and gender (shaped by social and cultural circumstances) carefully in order to avoid confusing both terms. Indicate if findings apply to only one sex or gender; describe whether sex and gender were considered in study design; whether sex and/or gender was determined based on self-reporting or assigned and methods used. Provide in the source data disaggregated sex and gender data, where this information has been collected, and if consent has been obtained for sharing of individual-level data; provide overall numbers in this Reporting Summary. Please state if this information has not been collected. Report sex- and gender-based analyses where performed, justify reasons for lack of sex- and gender-based analysis.*
Reporting on race, ethnicity, or other socially relevant groupings	*Please specify the socially constructed or socially relevant categorization variable(s) used in your manuscript and explain why they were used. Please note that such variables should not be used as proxies for other socially constructed/relevant variables (for example, race or ethnicity should not be used as a proxy for socioeconomic status). Provide clear definitions of the relevant terms used, how they were provided (by the participants/respondents, the researchers, or third parties), and the method(s) used to classify people into the different categories (e.g. self-report, census or administrative data, social media data, etc.) Please provide details about how you controlled for confounding variables in your analyses.*
Population characteristics	*Describe the covariate-relevant population characteristics of the human research participants (e.g. age, genotypic information, past and current diagnosis and treatment categories). If you filled out the behavioural & social sciences study design questions and have nothing to add here, write "See above."*
Recruitment	*Describe how participants were recruited. Outline any potential self-selection bias or other biases that may be present and how these are likely to impact results.*
Ethics oversight	*Identify the organization(s) that approved the study protocol.*

Note that full information on the approval of the study protocol must also be provided in the manuscript.

Field-specific reporting

Please select the one below that is the best fit for your research. If you are not sure, read the appropriate sections before making your selection.

☒ Life sciences ☐ Behavioural & social sciences ☐ Ecological, evolutionary & environmental sciences

For a reference copy of the document with all sections, see nature.com/documents/nr-reporting-summary-flat.pdf

Life sciences study design

All studies must disclose on these points even when the disclosure is negative.

Sample size	Specific a priori effect size calculation for the multi-modal analysis was not performed. A total of 40 mice were used across 4 independent infection experiments: Spatial transcriptomics and mass-spectrometry based lipidomics (n=8; 4 infected and 4 naïve), single-cell RNA-seq (n=12; 6 infected (3 matched mice from spatial transcriptomics and 3 from an independent experiment) mice plus 2 pools of 3 naïve mice each), flow-sorted proteomics (n=8 infected mice, from which CD172hi macrophages [n=8 samples] and CD172lo macrophages [n=5 samples] were obtained), and flow cytometry characterization of LPCAT2 and NOS2 expression in myeloid populations (n=7 naïve and n=8 infected). From flow sorted samples, due to low cell recovery post-sort in the CD172lo fraction, led to three unmatched CD172hi animals.

Data exclusions	While integrating Visium and mass spectrometry based imaging data certain pixels did not co-register. These pixels were excluded form analysis.<br><br>Lipid intensity clustering in some naïve samples displays geometric patterns, which we attribute to incomplete spatial coverage during positive ionization mode acquisition. This reflects the inherent technical challenges of co-registering multiple ionization modes across multiple tissue sections, where complete spatial coverage in positive ionization mode was not achieved in some regions of these samples. The largest effect is seen in the naive sample, N4. In addition, our analytical method mitigates this by guiding the downstream analysis using RNA clusters with lipid intensities incorporated as metadata.
Replication	All key experiments were replicated across multiple biological samples demonstrating reproducibility of findings
Randomization	Animals were not randomized into experimental groups; all mice used were age-matched controls or infected animals.
Blinding	Blinding was not employed during data acquisition or analysis due to clear experimental conditions (infected versus naïve mice), minimizing potential bias.

Reporting for specific materials, systems and methods

We require information from authors about some types of materials, experimental systems and methods used in many studies. Here, indicate whether each material, system or method listed is relevant to your study. If you are not sure if a list item applies to your research, read the appropriate section before selecting a response.

Materials & experimental systems

n/a	Involved in the study
☐	☒ Antibodies
☒	☐ Eukaryotic cell lines
☒	☐ Palaeontology and archaeology
☐	☒ Animals and other organisms
☒	☐ Clinical data
☒	☐ Dual use research of concern
☒	☐ Plants

Methods

n/a	Involved in the study
☒	☐ ChIP-seq
☐	☒ Flow cytometry
☒	☐ MRI-based neuroimaging

Antibodies

Antibodies used

1. NOS2Alexa Fluor 488 eBioscience 53-5920-82 (Clone CXNFT, 1:250 dilution)
2. F4/80 Alexa Fluor 594 BioLegend 123140 (Clone BM8, 1:250 dilution)
3. SIRPα/CD172a Alex a Fluor 647 BioLegend 144027 (Clone P84, 1:200 dilution)
4. LPCAT2 Coralite488ThermoFisher CL488-15082 (Polyclonal, 1:125 dilution)
5. Ly6G PerCP-Cy5.5 BioLegend 127616 (Clone 1A8, 1:100 dilution)
6. F4/80 FITC; or PE-Cy7BioLegend 123114 (Clone BM8, 1:100 dilution)
7. CD11b APC; or BV421 ThermoFisher 17-0112-82 (Clone M1/70, APC 1:200; BV421 1:20 dilution)
8. Ly6C BV421 BioLegend 128032 (Clone HK1.4, 1:20 dilution)
9. CD172a (SIRPα) PE ThermoFisher 12-1721-82 (Clone P84, 1:100 dilution)
10. CD3ε PE-Cy7 BioLegend 100319 (Clone 145-2C11, 1:100 dilution)
11. NOS2 PE-eFluor 610 ThermoFisher 61-5920-82 (Clone CXNFT, 1:300 dilution)
12. LPCAT2- (unconjugated) Invitrogen PA5-101406 (Rabbit IgG polyclonal, 1:500 dilution)
13. ApoE Alexa Fluor 594 Abcam ab310350 (Rabbit monoclonal, Clone EPR19392)

Validation

1. https://www.thermofisher.com/antibody/product/iNOS-Antibody-clone-CXNFT-Monoclonal/53-5920-82
2. https://www.biolegend.com/en-gb/search-results/alexa-fluor-594-anti-mouse-f4-80-antibody-9631?GroupID=BLG5319
3. https://www.biolegend.com/en-gb/products/alexa-fluor-647-anti-mouse-cd172a-sirpalpha-antibody-15378
4. https://www.thermofisher.com/antibody/product/LPCAT2-Antibody-Polyclonal/CL488-15082
5. https://www.biolegend.com/en-us/products/percp-cyanine5-5-anti-mouse-ly-6g-antibody-6116
6. https://www.biolegend.com/en-us/products/pe-cyanine7-anti-mouse-f4-80-antibody-4070
7. https://www.thermofisher.com/antibody/product/CD11b-Antibody-clone-M1-70-Monoclonal/17-0112-82
8. https://www.biolegend.com/en-us/products/brilliant-violet-421-anti-mouse-ly-6c-antibody-8586
9. https://www.thermofisher.com/antibody/product/CD172a-SIRP-alpha-Antibody-clone-P84-Monoclonal/12-1721-82
10. https://www.biolegend.com/en-us/products/pe-cyanine7-anti-mouse-cd3epsilon-antibody-1899
11. https://www.thermofisher.com/antibody/product/iNOS-Antibody-clone-CXNFT-Monoclonal/61-5920-82
12. https://www.thermofisher.com/antibody/product/LPCAT2-Antibody-Polyclonal/PA5-101406
13. https://www.abcam.com/en-us/products/primary-antibodies/alexa-fluor-594-apolipoprotein-e-antibody-epr19392-ab310350

Animals and other research organisms

Policy information about studies involving animals; ARRIVE guidelines recommended for reporting animal research, and Sex and Gender in Research

Laboratory animals	Mus musculus, C57BL/6J, female
Wild animals	n/a
Reporting on sex	Only female mice were used in this study to avoid potential variability due to sex-related differences in immune responses and disease progression in the model of visceral leishmaniasis for the spatial and single-cell integration study.<br><br>For validation experiments for proteomics and flow cytometry, both male and female mice were used.
Field-collected samples	n/a
Ethics oversight	UK Home Office

Note that full information on the approval of the study protocol must also be provided in the manuscript.

Plants

Seed stocks	*Report on the source of all seed stocks or other plant material used. If applicable, state the seed stock centre and catalogue number. If plant specimens were collected from the field, describe the collection location, date and sampling procedures.*
Novel plant genotypes	*Describe the methods by which all novel plant genotypes were produced. This includes those generated by transgenic approaches, gene editing, chemical/radiation-based mutagenesis and hybridization. For transgenic lines, describe the transformation method, the number of independent lines analyzed and the generation upon which experiments were performed. For gene-edited lines, describe the editor used, the endogenous sequence targeted for editing, the targeting guide RNA sequence (if applicable) and how the editor was applied.*
Authentication	*Describe any authentication procedures for each seed stock used or novel genotype generated. Describe any experiments used to assess the effect of a mutation and, where applicable, how potential secondary effects (e.g. second site T-DNA insertions, mosiacism, off-target gene editing) were examined.*

Flow Cytometry

Plots

Confirm that:

- [x] The axis labels state the marker and fluorochrome used (e.g. CD4-FITC).
- [x] The axis scales are clearly visible. Include numbers along axes only for bottom left plot of group (a 'group' is an analysis of identical markers).
- [x] All plots are contour plots with outliers or pseudocolor plots.
- [x] A numerical value for number of cells or percentage (with statistics) is provided.

Methodology

Sample preparation	Liver tissues from infected and uninfected mice were dissected and immediately processed into single-cell suspensions. Tissue was coarsely sliced in PBS supplemented with 2% fetal calf serum (FCS), digested enzymatically with collagenase and DNase at 37°C for 20 minutes, then digestion was halted with ice-cold RPMI containing FCS. Cells were strained through a 100µm mesh, lysed using ACK buffer to remove erythrocytes, and subsequently purified by centrifugation in 33% Percoll to remove hepatocytes. The resulting single-cell suspensions were resuspended in PBS with 0.05% BSA and kept on ice until flow cytometry staining.
Instrument	Beckman Coulter MoFlo Astrios with 4 laser lines (405, 488, 561 and 633 nm)
Software	Subsequent data analysis, gating adjustments, and visualizations were carried out using FlowJo software (10.10.0)
Cell population abundance	The cell sorting strategy targeted macrophage populations identified as CD3-CD11b+F4/80+ cells, further subdivided into CD172hi and CD172lo subsets. CD172hi cells represented approximately 68% and CD172lo cells about 24% of the total sorted macrophage population.
Gating strategy	Gating employed for sorted proteomics: The gating strategy involved sequential gates set to first identify leukocytes based on FSC/SSC profiles, followed by exclusion of doublets, dead cells, and CD3-positive T-cells. From this parent gate, macrophages were identified as CD11b+F4/80+ and further distinguished based on the intensity of CD172 (SIRPA) expression into CD172hi (LPCAT2-high expressing macrophages) and CD172lo populations for downstream proteomic analyses.<br><br>Gating employed for figure 8: Flow cytometry gating strategy for identifying liver myeloid cell populations. Sequential gating

shows: initial cell population based on forward/side scatter, (1) single cell discrimination, (2) live cells, (3a) CD11b+F4/80+ (strategy 1) Or (3b) CD11b+ cells, (4) neutrophil exclusion (CD11b+Ly6g-), and (5) final myeloid subpopulations based on F4/80 and Ly6c expression as strategy 2. Strategy 2 (bottom right) classifies myeloid cells into four populations: inflammatory monocytes, F4/80+Ly6c_high, transitional Ly6c_intermediate, and F4/80+Ly6c_low resident macrophages.

☒ Tick this box to confirm that a figure exemplifying the gating strategy is provided in the Supplementary Information.
